# Book Review: Food: The Chemistry of Its Components: 6^th^ Edition

**DOI:** 10.3389/fchem.2017.00065

**Published:** 2017-09-15

**Authors:** Sushilkumar A. Jadhav

**Affiliations:** ^1^Department of Chemistry, University of Torino Torino, Italy; ^2^NIS Research Centre, University of Torino Torino, Italy

**Keywords:** food chemistry, sugars, lipids, proteins, vitamins, minerals, water

Food is a topic of interest and the chemical information about food components helps to know “what exactly are the structures, properties, and functions of different food components?” Various books describing the chemistry of food components are published recently (Sikorski, 2006; Velisek, 2014; Cheung and Mehta, 2015). Some of these books are research article or chapter collection books edited by specialists. The title book by Tom Coultate is one of the most popular books and best seller from RSC books. This book is the 6^th^ successive edition of the first book published in 1984 (Coultate, 1984). Although the target readers of the book are chemists, it is an excellent reference book also for biologists, biochemists, agriculture, and food chemistry students and teachers. The readers, even with little organic or general chemistry background can easily understand it.

The important features of the book are that it is organized chapter wise in a logical order and simple language is used to explain the chemical structures of food components, their properties, and role in the metabolism. The book contains 13 chapters which describe the different food components. The chapter (1) includes a brief introduction of the topic and the chapters (2) sugars, (3) polysaccharides, (4) lipids, (5) proteins, and (8) vitamins describe the essential food components. The chapters (6) colors, (7) flavors, (9) preservatives, (10) undesirable things in food, and (11) minerals are devoted to the chapter title topics. The important addition in this edition of the book is the chapter 12 “enzymes” which contains basic information of enzymes with their actions, their activity in food materials and applications. The last chapter (13^th^) is “water” which contains the information about interactions of water with various food components and the quantitative determination of water in food. The general importance of the book can be guessed by two examples of information mentioned in chapter 2, sugars, where the percentage of lactose in different milk sources (human, cow, goat, sheep, reindeer, etc.) and dairy products (butter, cheese, etc.) and total percentage of sugars in different breads, vegetables, and fruits are reported. The latest information sources are cited in the book and a collective reference section named “further reading” is provided at the end of each chapter which includes the list of books and reviews. Although the collective information provided about food components in the book is basic and it can be found in various sources, the information in the book is illustrated in an effective way with many structures, tables and figures which makes it easy to understand.

The readability stats and reading ease level showed that the book can be easily understood by starting level science students which is another great feature of the book. As most of the science graduation and master courses contain a section dedicated to food and its components, this book is an excellent reference text-book for those courses. The book is an important and updated source of collective information about the chemistry of food and therefore it is highly recommended.

## Author contributions

The author confirms being the sole contributor of this work and approved it for publication.

### Conflict of interest statement

The author declares that the research was conducted in the absence of any commercial or financial relationships that could be construed as a potential conflict of interest.
